# Differences in awareness of positive and negative age-related changes accounting for variability in health outcomes

**DOI:** 10.1007/s10433-021-00673-z

**Published:** 2022-02-04

**Authors:** Serena Sabatini, Obioha C. Ukoumunne, Allyson Brothers, Manfred Diehl, Hans-Werner Wahl, Clive Ballard, Rachel Collins, Anne Corbett, Helen Brooker, Linda Clare

**Affiliations:** 1 REACH, College of Medicine and Health, University of Exeter, South Cloisters, St Luke’s Campus, Exeter, EX12LU UK; 2 NIHR Applied Research Collaboration South West Peninsula (PenARC), University of Exeter, Exeter, UK; 3 College of Health and Human Sciences, Colorado State University, Fort Collins, CO USA; 4 Institute of Psychology, Heidelberg University, Heidelberg, Germany; 5 Ecog Pro Ltd., Bristol, UK

**Keywords:** AARC, Subjective cognitive decline, Subjective ageing, Latent profile analysis

## Abstract

Higher awareness of positive age-related changes (AARC gains) is related to better mental health, whereas higher awareness of negative age-related changes (AARC losses) is related to poorer mental and physical health. So far perceived gains and losses have been explored separately, but people report gains and losses concurrently in varying degrees, and different profiles of gains and losses may be differentially associated with health. We identified profiles of gains and losses and explored whether different profiles differed in physical, mental, and cognitive health. We used cross-sectional data from the PROTECT study (*N* = 6192; mean (SD) age = 66.1 (7.0)). Using latent profile analysis, a four-class solution showed the best model fit. We found that 45% of people perceived many gains and few losses (Class 1); 24% perceived moderate gains and few losses (Class 2); 24% perceived many gains and moderate losses (Class 3); 7% perceived many gains and many losses (Class 4). Analysis of variance and Chi-squared tests showed that Class 1 had relatively better physical, mental, and cognitive health, followed by Classes 2, 3, and 4. Experiencing one’s ageing to a high degree as gain may be related to better health only when individuals interpret ageing as involving low levels of loss across several life domains. Risk in terms of poorer health emerged in those who perceived high losses. Considering gains and losses in parallel, rather than separately, may lead to a more fine-tuned understanding of relations with health.

**Supplementary Information:**

The online version contains supplementary material available at 10.1007/s10433-021-00673-z.

## Introduction

Self-perceptions of ageing comprise an important part of the ageing process and provide valuable information about current and future levels of health (Westerhof et al. 2014). Individuals’ self-perceptions of ageing can even be more informative of future health than scores obtained through objective measures of health (Sargent-Cox et al. 2012). Individuals who perceive their ageing more positively tend to have better physical, mental, and cognitive health compared to those individuals with more negative self-perceptions of ageing (Boeder and Tse 2021; Brothers et al. 2020; Levy et al. 2018; Sabatini et al. 2021b; Siebert et al. 2016, 2018; Wurm et al. 2007). This association may be due to individuals with positive self-perceptions of ageing being more engaged in health-related behaviours (Meisner et al. 2013; Wurm et al. 2010). Self-perceptions of ageing can therefore be useful to identify those individuals that could benefit the most from health-promoting interventions.

A limitation of many existing studies exploring the association between how individuals rate their own ageing and their actual health is that they used uni-dimensional measures (Diehl et al. 2014), such as asking individuals to report the age they commonly feel (subjective age; Barrett 2003). However, when evaluating their own ageing, people often take into account their different experiences across several life domains (e.g. mental and physical domains; Steverink et al. 2001; Voss et al. 2018). A further limitation of existing evidence is that it relies on constructs (e.g. attitudes towards own ageing; Lawton 1975) that assess self-perceptions of ageing in a uni-dimensional way. As a consequence, an individual can report either positive (gains) or negative (losses) self-perceptions of ageing, but not both. However, individuals’ evaluations of their own ageing are often mixed and can include varying amounts of gains and losses (Miche et al. 2014). On the one hand, older individuals often experience unwelcome changes that, for instance, can be the result of disability, complex co-morbidities, and chronic health conditions. These changes can have a negative impact on individuals’ self-perceptions of ageing (Barnett et al. 2012; Kingston et al. 2018; Levy 2017; Royall et al. 2007). On the other hand, older individuals can contribute greatly to the workforce, providing knowledge and experience, and engage in volunteer activity. This, together with the experience of valuable social relations, increased leisure time, and accumulated knowledge and life experience, may foster positive self-perceptions (Carstensen 1993; Carstensen et al. 2011; Steverink et al. 2001; Timmer et al. 2002). In summary, when exploring the association of self-perceptions of ageing with health, it is important to consider perceptions of both developmental gains and losses and their possible coexistence in multiple life domains.

Several scales assessing both positive and negative self-perceptions of ageing exist; major examples are the Attitudes to Aging Questionnaire (Laidlaw et al. 2007), the Aging Perception Questionnaire (Barker et al. 2007), the Aging-related Cognitions scales (Steverink et al. 2001), and the Expectations Regarding Aging scales (Sarkisian et al. 2002, 2005b). These scales enabled researchers to study the separate associations that perceived developmental gains and perceived developmental losses have with health outcomes (Steverink et al. 2001). Although for some of these scales both a full (ERA: 38 items; APQ: 32 items; AAQ: 24 items; AgeCog: 12 items) and a short form (AAQ: Laidlaw et al. 2018; ERA: Sarkisian et al. 2005a; APQ: Sexton et al. 2014) are available; the short versions have rarely been used in their full dimensionality in the previous research (e.g. subscale on social loss not considered, Wolff et al. 2018; Wurm et al. 2007). Hence, research on the multi-dimensionality of self-perceptions of ageing remains somewhat incomplete. Moreover, some of the scales seem to overlap with other major constructs (for instance the APQ captures control beliefs) or mix “change” with “loss” and “growth” (AAQ).

Limitations like these have been overcome to some extent with the introduction of the awareness of age-related changes (AARC) concept which captures “*a person’s state of awareness that his or her behaviour, level of performance, or way of experiencing life has changed as a consequence of having grown older*” (Diehl and Wahl 2010; p. 342) and the introduction of the AARC assessment in an ultra-short version (AARC-10 SF) for use in survey research (Kaspar et al. 2019). The AARC-10 SF is a two-dimensional construct comprising awareness of positive (AARC gains) and negative (AARC losses) age-related change with the five items per gain and loss dimension representing five life domains (health and physical functioning, cognitive functioning, interpersonal relationships, socio-cognitive and socio-emotional functioning, and lifestyle/engagement). These life domains, originally developed as conceptual guidance (Diehl and Wahl 2010), were confirmed by the findings of a semi-structured intensive diary study in 70- to 88-year-old adults (Miche et al. 2014). Moreover, AARC assumes that gains and losses can occur simultaneously. AARC is conceptually anchored in lifespan developmental psychology that builds its conceptualisation of human development on the coexistence of developmental gains and losses (Baltes 1987; Baltes et al. 2006) and how self-awareness of developmental change may be a precondition for adaptation to age-related changes (Brandtstädter and Rothermund 2002). As has also been found, the perception of AARC gains is quite independent from the perception of AARC losses and correlations between perceived gains and losses were found to be below *r* = 0.15 (Kaspar et al. 2019).

Self-reported levels of AARC gains and losses across the five AARC life domains explain variability in health-related outcomes (Sabatini et al. 2020a). Higher AARC losses across the five AARC life domains are associated with worse physical and functional health (Brothers et al. 2019; Sabatini et al. 2020c), whereas higher AARC gains and AARC losses across the five AARC life domains are associated with better and worse mental health, respectively (Brothers et al. 2016; Dutt et al. 2016a). Moreover, individuals having fewer AARC gains and/or more AARC losses across the five AARC life domains are less likely to use self-regulatory strategies (Dutt et al. 2016b; Wilton-Harding and Windsor 2021), to follow a healthy lifestyle (Brothers and Diehl 2017), and to have high self-efficacy (Dutt and Wahl 2018), which can also contribute to poor physical and mental health. Finally, higher levels of AARC losses across the five AARC life domains are correlated with worse cognitive performance (Sabatini et al. 2021a; Zhu and Neupert 2020). Only one cross-sectional study found that higher levels of AARC gains are related to poorer cognitive performance; however, associations were small to negligible (Sabatini et al. 2021a). As health is more strongly associated with AARC losses than AARC gains (Sabatini et al. 2020a), individuals’ current cognitive functioning may be captured by perceived losses, irrespective of the coexistence of perceived gains. Evidence on AARC is limited to the study of the separate associations of gains and losses with health; as a consequence, how the coexistence of different levels of gains and losses relates to health indicators is unknown.

Existing research so far has reported on average population levels for AARC gains and AARC losses. In the USA, Germany, and UK, on average, middle-aged and older adults reported “quite a bit” of AARC gains and “a little bit” of AARC losses across the five AARC life domains (Brothers et al. 2019; Kaspar et al. 2019; Sabatini et al. 2020b). It may be that within a population, there are different profiles of individuals having varying levels of AARC gains and losses. Indeed, among middle-aged and older individuals, there is great variability in levels of physical, mental, and cognitive health (Andreas et al. 2017; Brayne et al. 2001; Deary et al. 2009; Evandrou and ESRC SAGE Research Group 2005; Health and Social Care Information Centre 2007) and this may be reflected in individuals’ perceptions of AARC and in the combination of perceived levels of AARC gains and losses. By taking an exploratory approach, with the current study, we aim to identify the number and types of profiles of AARC gains and AARC losses using a large sample of UK individuals aged 50 and over. We also aim to examine how participants’ profiles of AARC gains and AARC losses relate to a broad range of health indicators including physical, mental, and cognitive functioning.

## Methods

### Study design and participants

We used cross-sectional data collected through the ongoing PROTECT (https://www.protectstudy.org.uk) study in 2019. Individuals were eligible to participate in the PROTECT study if they were UK residents, English speakers, aged 50 years or over, had access to the Internet, and did not have a clinical diagnosis of dementia at the point of recruitment. In PROTECT, participants were recruited through national publicity and via existing cohorts of older adults (Brains for Dementia Research; Exeter 10,000; Join Dementia Research). Potential participants were enrolled through the PROTECT study website and provided informed consent. As part of the 2019 annual assessment, PROTECT participants were invited to complete additional measures on self-perceptions of ageing and health. Analyses for this study are based only on those participants who completed the additional measures between 1st January 2019 and 31st March 2019. The PROTECT study obtained ethical approval from the London Bridge NHS Research Ethics Committee and Health Research Authority (Ref: 13/LO/1578). Ethical approval for the data analysis was sought through the ethics committee at the University of Exeter, School of Psychology (Application ID: eCLESPsy000603 v1.0).

The study sample comprised 6,192 participants. The mean (standard deviation (SD); range) age was 66.1 years (7.0 years; 51 to 95 years) and 76.0% were women. Further demographic characteristics are reported in Table 1. Only 0.37% of participants reported having mild cognitive impairment (MCI). Based on the cognitive task scores, 5.6% of participants might experience age-associated cognitive decline (as they scored more than 1 SD below the mean sample score in two or more cognitive tasks) and 1.7% might have MCI (as they scored more than 1.5 SD below the mean sample score in two or more cognitive tasks).

**Table 1:** Descriptive statistics of demographic variables and main study variables

Variables	
Age in years, Mean (SD; Range)	66.1 (7; 51.4 to 95.9)
Female, %	75.9
Marital status, %	
Married	67.4
Civil partnership	0.5
Co-habiting	5.9
Widowed	7.4
Separated	1.7
Divorced	11.0
Single	6.2
Education level, %	
Secondary education	13.7
Post-secondary education	11.3
Vocational qualification	20.1
Undergraduate degree	33.8
Post-graduate degree	17.3
Doctorate	3.9
Currently employed, %	
Employed full-time	15.8
Employed part-time	15.7
Self-employed	9.5
Retired	56.7
Unemployed	2.4
AARC gains, %	
Not at all	0.1
A little bit	5.1
Moderately	22.2
Quite a bit	47.6
Very much	25.0
AARC losses, %	
Not at all	3.8
A little bit	61.0
Moderately	28.7
Quite a bit	5.7
Very much	0.8
Digit span, Mean (SD)	7.6 (1.5)
Paired associate learning, Mean (SD)	4.7 (0.9)
Grammatical reasoning, Mean (SD)	37.4 (10.6)
Self-ordered search, Mean (SD)	7.7 (2.6)
Self-rated health, %	
Poor	2.0
Fair	12.9
Good	54.5
Excellent	30.6
Functional ability, Mean (SD)	0.2 (0.8)
Depressive symptoms, Mean (SD)	11.4 (2.9)
Anxiety symptoms, Mean (SD)	8.4 (2.4)

Sample size ranged from 5811 to 6192. Secondary education—GCSE/O-Levels. Post-Secondary education—College, A-Levels, NVQ3 or below, or similar. Vocational qualification—Diploma, Certificate, BTEC, NVQ 4 and above, or similar. Undergraduate Degree—BA, BSc, or similar. Post-graduate Degree—MA, MSc, or similar. Doctorate—PhD. AARC gains—Total score on the AARC gains subscale from the AARC-10 SF. AARC losses—Total score on the AARC losses subscale from the AARC-10 SF. Digit span—Computerised cognitive task assessing verbal working memory. Paired associate learning—Computerised cognitive task assessing visual episodic memory. Grammatical reasoning—Computerised cognitive task assessing verbal reasoning. Self-ordered search—Computerised cognitive task assessing spatial working memory. IADL—Instrumental activities of daily living. Depressive symptoms—Score on the Patient Health questionnaire (PHQ-9). Anxiety symptoms—Score on the Generalized Anxiety Disorder questionnaire (GAD-7)

## Measures

### Socio-demographic variables

Participants reported their age, sex, marital status (*married*, *in a civil partnership*, *co-habiting*, *unmarried*, *divorced*, *separated*, and *widowed*), education level (*secondary education, post-secondary education, vocational qualifications, undergraduate degrees, post-graduate degrees, doctorates*), and current employment (*employed full-time, employed part-time, self-employed, retired, unemployed*).

### Awareness of age-related change (AARC)

The AARC-10 SF (Kaspar et al. 2019) contains ten items (reported in Supplementary Table 1); five assessing AARC gains and five assessing AARC losses. Each item per gain and loss dimension represents one out of five life domains (health and physical functioning, cognitive functioning, interpersonal relationships, socio-cognitive and socio-emotional functioning, and lifestyle/engagement; see again Miche et al. 2014). All items start with the same stem: “With my increasing age, I realise that…”. Respondents rate how much each item applies to them on a five-point Likert scale (from “not at all” (1) to “very much” (5)). Scores can be obtained for the AARC gains and AARC losses subscales by summing items that fall into the respective scales. Scale scores range from a minimum of five to a maximum of 25; higher scores indicate higher levels of AARC. In the current study sample, Cronbach’s *α* values (internal consistency) for the AARC gains and the AARC losses scales are 0.76 and 0.79, respectively (Sabatini et al. 2020b).

### Indicators of physical and functional health

Lawton’s Instrumental Activities of Daily Living Scale (IADL; Lawton and Brody 1969) is a seven-item instrument assessing everyday functional status. Each item describes an activity (e.g. preparing meals). Respondents have to rate how difficult they find performing the activity (0 = “no difficulty”, 1 = “some difficulty”, and 2 = “great difficulty”). The total score ranges from a possible 0 to 14, with higher scores indicating greater functional difficulty. Inter-rater reliability for the IADL scale, quantified using Pearson’s correlation, was 0.85 (Lawton and Brody 1969). In the current study sample, Cronbach’s *α* value for the IADL scale is 0.79.

Self-rated health was assessed with a single-item question (taken from the SF-36; Ware and Sherbourne 1992) asking participants to rate their own health on a four-point scale (Excellent, good, fair, poor).

### Mental health

The Patient Health Questionnaire-9 (PHQ-9; Kroenke et al. 2001) is a nine-item scale capturing depressive symptoms over the previous two weeks. Respondents are asked to indicate how frequently they experience each symptom on a 4-point scale (from “not at all” (1) to “nearly every day” (4)). The total score is the sum of the item scores and can range from a possible 9 to 36; higher scores indicate the presence of more depressive symptoms. The PHQ-9 has excellent internal consistency with Cronbach’s *α* coefficient of 0.84 in the normative sample (Kroenke et al. 2001) and of 0.76 in the current study sample.

The Generalized Anxiety Disorder-7 (GAD-7; Spitzer et al. 2006) is a seven-item measure asking respondents to indicate the frequency of occurrence of a list of symptoms of generalized anxiety disorder on a 4-point scale (from “not at all” (1) to “nearly every day” (4)). The scale score is the sum of the item scores and ranges from a possible 7 to 28; higher scores indicate greater presence of anxiety symptoms. The GAD-7 is an internally consistent measure with a Cronbach’s *α* of 0.92 in the normative sample (Spitzer et al. 2006) and of 0.86 in the sample of the current study.

### Cognitive functioning

Cognitive function was measured with the PROTECT Cognitive Test Battery (Corbett et al. 2015; Hampshire et al. 2012; Huntley et al. 2018) which includes four tasks: Digit Span; Paired Associate Learning; Grammatical Reasoning; and Self-ordered Search. For each task, a summary score can be obtained by subtracting the number of errors from the number of correct answers; a higher score indicates better performance. For Digit Span, the summary score can range from 0 to 20. For Paired Associate Learning, the summary score can range from 0 to 16. For Grammatical Reasoning, the summary score is also obtained by subtracting the number of errors from the number of correct answers, but the score has no upper or lower limit due to the fact that respondents can make attempts on as many trials as they can in the available time (three minutes). Finally, the summary score for the Self-ordered Search Task can range from 0 to 20.

## Analyses

To explore whether the population can be divided into classes of individuals characterised by different profiles of levels of AARC gains and losses, we conducted latent profile analysis, using Mplus software (Muthén and Muthén 2017). We fit the latent profile models based on manifest variables representing responses to the 10 items of the AARC-10 SF (5 gain items and 5 loss items). The 10 items assessing AARC were treated as continuous manifest variables. To identify the model with the optimal number of classes, we fit a two-class model and systematically increased the number of classes by 1 until adding more classes no longer resulted in an improvement in model fit and did not compromise the parsimony of the model. The main criteria used to identify the best fitting model were the Vuong-Lo-Mendell-Rubin and Lo-Mendell-Rubin adjusted likelihood ratio tests; we also report Akaike’s information criterion (AIC), the Bayesian information criterion (BIC) and the entropy statistic (Nylund et al. 2007). The best fitting model is the one which has a low value on the information criterion while being parsimonious in the number of identified classes. Having identified the best fitting model, we reported estimates of the percentage of the population falling in each class and estimates of the mean and standard deviation for each of the 10 AARC items for each class. The latter estimates were the basis for assigning names that characterise the profile of responses in each class.

Analysis of variance and Chi-squared tests were conducted to compare physical, functional, mental (depressive and anxiety symptoms), and cognitive health (scores on tasks assessing digit span, paired associate learning, grammatical reasoning, and self-ordered search) across the classes identified in the latent profile analysis. For the analysis of variance comparing physical, functional, and mental health and cognitive functioning, we fitted two models: one unadjusted and one adjusted for the effects of age, sex, marital status, education level, and current employment. We adjusted for these demographic variables as they may all be related to levels of mental, physical, and cognitive health in older age (Alavinia and Burdorf 2008; Alexopoulos 2005; Anstey et al. 2017; Banazak 1997; Herd et al. 2007; Hughes and Waite 2009; Weyerer et al. 2013). For these analyses, study participants were allocated to the class for which they had the greatest probability of membership. For the results from the analyses of variance, the effect size was calculated using eta squared (*η*^2^). We interpreted effect sizes for the eta squared between 0.01 and 0.05 as small, between 0.06 and 0.13 as moderate and of 0.14 or above as large (Cohen 1988). The analyses were carried out using STATA version 16 (StataCorp 2017).

## Results

### Descriptive analyses

A high proportion of participants perceived their health as good (54.6%) or excellent (30.1%) and reported no functional difficulties. The levels of depression (mean (SD) = 11.4 (2.9)) and anxiety (mean (SD) = 8.4 (2.4)) are similar to those in the general population of older adults (World Health Organization 2020). The majority of participants perceived “a little bit” (61.0%) or a “moderate” (28.7%) level of AARC losses and “moderate” (22.2%), “quite a bit” (47.6%), or “very much” (25%) AARC gains. Further details of descriptive analyses are reported in Table 1.

### Pattern of AARC gains and losses profiles

The first objective of this study was to identify the classes that represent different profiles of AARC gains and losses. Among the fitted models (two-class, three-class, four-class, and five-class models), we selected the four-class model as the best, based on the results of the Vuong-Lo-Mendell-Rubin and Lo-Mendell-Rubin adjusted likelihood ratio tests (see Table 2 for goodness of fit statistics anf entropy). The entropy statistic for the four-class model was 0.813, suggesting that the classes are fairly well defined. In the selected model, participants in Class 1 reported *many gains and few losses* (45%); participants in Class 2 reported *moderate gains and few losses* (24%); participants in Class 3 reported *many gains and moderate losses* (24%); and participants in Class 4 reported *many gains and many losses* (7%). The means of the AARC items are shown for each class in Table 3. Based on allocating the sample participants to the class to which they had the highest probability of belonging, 2,833 participants were allocated to Class 1, 1,493 to Class 2, 1,420 to Class 3, and 446 to Class 4.

**Table 2:** Goodness of fit indices for the four latent profile models

Model	Akaike’s information criterion (AIC)	Bayesian information criterion (BIC)	Likelihood ratio test *p* value		Entropy
			Vuong–Lo–Mendell–Rubin	Lo–Mendell–Rubin adjusted	
Two-class model	164,047.3	164,256	< 0.0001	< 0.0001	0.897
Three-class model	157,715.2	157,997.9	< 0.0001	< 0.0001	0.868
Four-class model	156,061.9	156,418.7	0.0001	0.0001	0.813
Five-class model	154,819.7	155,250.5	0.17	0.17	0.780

*LR* Likelihood ratio

**Table 3:** Comparison of physical and mental health, and cognitive functioning across the four profiles of AARC gains and losses

Variables	Class 1	Class 2	Class 3	Class 4	Unadjusted		Adjusted	
					*p* value	*η*^2^	*p* value	*η*^2^
Depressive symptoms, mean (SD)	10.8 (2.3)	11.4 (2.8)	11.9 (3.1)	13.8 (4.3)	< .0001	0.07	< .0001	0.04
Anxiety symptoms, mean (SD)	8.1 (2.1)	8.3 (2.2)	8.7 (2.8)	9.6 (3.3)	< .0001	0.03	< .0001	0.02
Functional health, mean (SD)	0.01 (0.4)	0.1 (0.7)	0.2 (0.9)	0.8 (1.8)	< .0001	0.06	< .0001	0.03
Perceived health, %								
Poor	0.5	0.8	2.2	15.5	< .0001			
Fair	5.9	11.7	20.0	38.1				
Good	51.6	58.9	61.1	36.1				
Excellent	42.0	28.4	16.7	9.4				
Digit span, mean (SD)	7.7 (1.6)	7.7 (1.5)	7.5 (1.5)	7.1 (1.5)	< .0001	0.01	.0008	0.01
Paired associate learning, mean (SD)	4.8 (0.9)	4.8 (0.9)	4.6 (0.9)	4.5 (0.9)	< .0001	0.01	.0002	0.01
Self-ordered search, mean (SD)	7.8 (2.6)	7.9 (2.6)	7.4 (2.6)	7.0 (2.9)	< .0001	0.01	.0002	0.01
Grammatical reasoning, mean (SD)	38.1 (10.3)	38.5 (10.5)	36.3 (10.2)	33.4 (11.4)	< .0001	0.02	.0005	0.02

In Class 1, the sample size ranged from 2820 to 2833; in Class 2, the sample size ranged from 1487 to 1493; in Class 3, the sample size ranged from 1413 to 1420; in Class 4, the sample size ranged from 442 to 446. Participants in Class 1 reported many AARC gains and few AARC losses. Participants in Class 2 reported moderate gains and few losses. Participants in Class 3 perceived many gains and moderate losses. Participants in Class 4 perceived many gains and many losses. Depressive symptoms—Score on the Patient Health questionnaire (PHQ-9). Anxiety symptoms—Score on the Generalized Anxiety Disorder questionnaire (GAD-7). Functional health—Total score on the Instrumental activities of daily living scale. Digit span—Computerised cognitive task assessing verbal working memory. Paired associate learning—Computerised cognitive task assessing visual episodic memory. Self-ordered search—Computerised cognitive task assessing spatial working memory. Grammatical reasoning—Computerised cognitive task assessing verbal reasoning. *η*^2^—Eta squared is the effect size. Adjusted analyses include age, sex, marital status, education level, and current employment

**Fig. 1:**
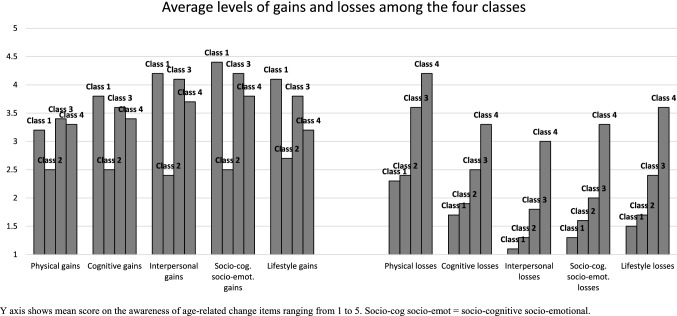
Mean scores for each AARC item by class membership. *Y* axis shows mean score on the awareness of age-related change items ranging from 1 to 5. Socio-cog socio-emot = socio-cognitive socio-emotional

Figure 1 and Supplementary Table 2 present the mean gains and losses scores reported in each of the five life domains captured by the multidimensional AARC construct, separately for each class. For all four classes, physical losses ranked higher than losses in other domains; not surprisingly, physical losses were the highest in Classes 3 and 4 compared to Classes 1 and 2. However, all four classes reported losses across all life domains which suggests that the pattern of overall findings was not driven solely by physical-related losses.

### Differences in health outcomes among the four classes of AARC gain and loss profiles

Using analysis of variance tests, we found that the four classes differed in mean levels of functional health, depressive symptoms, anxiety symptoms, and scores obtained on the cognitive tasks digit span, paired associate learning, grammatical reasoning, and self-ordered search (all *p*’s < 0.001; Table 3). The class with the best physical and mental health, and cognitive functioning was the one with “many gains and few losses” (Class 1), followed by the classes with “moderate gains and few losses” (Class 2), “many gains and moderate losses” (Class 3), and “many gains and many losses” (Class 4; Table 3). Differences in health outcomes among classes remained after controlling for demographic variables. Using Chi-squared tests, we found that the four classes were characterised by different proportions of participants who rated their health as poor (*p* < 0.0001; Table 3). The class of participants with “many gains and few losses” (Class 1) perceived their health most positively, followed by those who reported “moderate gains and few losses” (Class 2), “many gains and moderate losses” (Class 3), and “many gains and many losses” (Class 4).

## Discussion

Using a large sample of individuals aged over 50 in the UK, this is the first study to identify profiles of AARC gains and losses and showing how different profiles relate to participants’ physical and mental health, and cognitive functioning. We identified four profiles (Class 1 reported *many gains and few losses*; Class 2 reported *moderate gains and few losses*; Class 3 reported *many gains and moderate losses*; and Class 4 reported *many gains and many losses*), highlighting the common combinations of AARC gains and losses that individuals experience. The four classes differed in terms of physical and mental health, and cognitive functioning, although the size of the effects for cognitive functioning was small. Overall, the “many gains and many losses” class had the worst physical and mental health, and cognitive functioning, followed by the classes with “many gains and moderate losses”, “moderate gains and few losses”, and “many gains and few losses”. This pattern of results suggests that considering the coexistence of gains and losses is important for a comprehensive understanding of how perceived AARC relates to health.

In this sample, participants in the four groups perceived either moderate or many positive age-related changes; what differs the most among classes is the level of perceived negative age-related changes. Out of four classes, one perceived moderate levels of AARC losses and one perceived high levels of AARC losses. These two classes together only accounted for 31% of the population which is in line with previous evidence collected with multidimensional measures of self-perceptions of ageing. Moreover, there was no class reporting low AARC gains and high AARC losses, showing that older individuals tend to report more positive than negative self-perceptions of ageing, or at least as many positive as negative self-perceptions of ageing (e.g. Steverink et al. 2001). Our findings are, however, in contrast to existing literature on age stereotypes reporting that, for example, the majority of individuals have negative attitudes towards ageing (Levy 2017) and that the general public usually holds negative views of ageing, including a number of misconceptions about the ageing process (Diehl et al. 2020). Thus, the findings of the present study emphasise that individuals view their own ageing more positively than what is known from studies on age stereotypes. This discrepancy may be due to attitudes towards ageing capturing individuals’ generalised beliefs about ageing, whereas AARC captures what people have experienced as they grow older. It may be that many individuals have negative expectations about ageing that are not always met and, hence, do not come into play when evaluating their own ageing (Rothermund and Brandtstädter 2003). However, the small proportion of participants reporting moderate or many levels of AARC losses may also be due to our sample including individuals that on average had good health.

The better physical and mental health, and cognitive functioning in the group experiencing many gains and few losses extends literature reporting the separate associations between more AARC gains and better physical and mental health and between fewer AARC losses and better physical and mental health, and cognitive functioning (Sabatini et al. 2020a, 2021a). The class having “many gains and few losses” had better physical and mental health, and cognitive functioning than the class experiencing “moderate gains and few losses”, suggesting that the presence of gains may be related to higher levels of physical and mental health, and cognitive functioning. However, the class reporting many gains but also many concomitant losses showed the worst health status. Hence, higher AARC gains may be related to better health only when accompanied by low levels of AARC losses.

A study of an intervention aiming to increase engagement in health-related behaviours (e.g. physical activity) by including a component targeting views of ageing in addition to a behavioural component resulted in higher levels of perceived gains, reduced levels of perceived losses, and increased engagement in physical activity (Brothers and Diehl 2017). As study results suggest that individuals experiencing many losses are likely to perceive their health as poor, irrespective of whether they experience high or low levels of AARC gains, people who perceive many AARC losses may, therefore, benefit from interventions similar to the one implemented by Brothers and colleagues (2017).

## Limitations

The study has several limitations. First, as the sample included mainly white participants, women, individuals who were married (or in a civil partnership or co-habiting), well-educated, and in good health; results cannot be generalised to the broader population of middle-aged and older individuals. Moreover, as the mean age of the sample was 66.1 years; results may not be generalised to very old adults. For instance, it may be that among very old individuals, some experience high AARC losses and few AARC gains.

The samples are representative of the UK older population (e.g. English Longitudinal Study of Ageing, ELSA, https://www.elsa-project.ac.uk) and have a similar age distribution and proportion of married individuals; however, compared to ELSA, the PROTECT cohort includes better-educated individuals and a higher proportion of women (Taylor et al. 2007; Steptoe et al. 2013). Moreover, individuals were self-nominated to be part of the PROTECT study. Second, the cognitive tasks were not completed on the same day on which participants completed the AARC questionnaire but within two months of that date. This is a limitation given the daily variation in scores on the cognitive tasks (Huntley et al. 2018). However, giving participants the opportunity to complete the objective cognitive tasks on a separate day from the remaining measures decreased participants’ burden and increased the likelihood of collecting accurate answers. Third, mental and physical health were assessed through self-report measures, hence recall bias may have occurred (Althubaiti 2016). Moreover, poor self-reported health can in some cases be an indicator of poor mental health rather than of poor physical health (Schnittker 2005).

## Conclusions

This was the first study exploring the potential coexistence of AARC gains and losses and identifying which profiles of AARC gains and losses were common in the population. Among middle-aged and older individuals with above average perceived physical health and intact cognitive abilities, there were different profiles of coexistence of perceived AARC gains and losses. Most frequently, individuals perceived many age-related gains and few age-related losses, whereas the experience of both many gains and losses was the least frequent. This was also the first study exploring whether different profiles of AARC gains and losses are related to physical, mental, and cognitive health. Profiles with different combinations of AARC gains and losses differed with respect to physical, mental, and cognitive health, suggesting that assessing the coexistence of gains and losses is important when relating AARC to health.

## Supplementary Information

Below is the link to the electronic supplementary material.

Supplementary file1 (DOCX 16 KB)

## Data Availability

This study was conducted using secondary data collected as part of the PROTECT ongoing study. PROTECT data are available to investigators outside the PROTECT team after request and approval by the PROTECT Steering Committee. Data for the AARC-10 SF questionnaire will be available from May 2022.
