# Supplementary material for: Differences in awareness of positive and negative age-related changes accounting for variability in health outcomes
Source: Eur J Ageing. 2022 Feb 4;19(4):1087–97. doi: 10.1007/s10433-021-00673-z (PMC9729481; doi:10.1007/s10433-021-00673-z)
Supplement: Supplementary file 1 — Supplementary file1 (DOCX 16 KB) [file 10433_2021_673_MOESM1_ESM.docx]

**Supplementary Table 1. AARC-10 SF items**

| AARC-10 SF items | | |
| --- | --- | --- |
| With my increasing age, I realise that… | | |
| Scale | AARC domain^a^ | Items |
| AARC GAINS | PHYS | …I pay more attention to my health |
|  | COG | …I have more experience and knowledge to evaluate things and people |
|  | INT | …I appreciate relationship and people much more |
|  | SCSE | …I have a better sense of what is important for me |
|  | LIFE | …I have more freedom to live my days the way I want |
| AARC LOSSES | PHYS | … I have less energy |
|  | COG | …my mental capacity is declining |
|  | INT | …I feel more dependent on the help of others |
|  | SCSE | …I find it harder to motivate myself |
|  | LIFE | …I have to limit my activities |

Note: ^a^AARC domain abbreviations: PHYS= Health and physical functioning; COG = Cognitive functioning; INT = Interpersonal relations; SCSE = Social-cognitive and social-emotional functioning; LIFE = Lifestyle and engagement. AARC GAINS= Awareness of positive age-related changes. AARC LOSSES= Awareness of negative age-related changes.

**Supplementary Table 2. Mean (95% CI) for each AARC item by class membership**

|  | Class 1  (many gains and few losses) | Class 2  (moderate gains and few losses) | Class 3  (many gains and moderate losses) | Class 4  (many gains and many losses) |
| --- | --- | --- | --- | --- |
|  | Mean (95% CI) | Mean (95% CI) | Mean (95% CI) | Mean (95% CI) |
| AARC gains | | | | |
| Physical gains | 3.21 (3.16 to 3.25) | 2.48 (2.42 to 2.53) | 3.42 (3.36 to 3.48) | 3.34 (3.24 to 3.44) |
| Cognitive gains | 3.77 (3.73 to 3.81) | 2.46 (2.41 to 2.51) | 3.62 (3.56 to 3.68) | 3.36 (3.26 to 3.45) |
| Interpersonal gains | 4.20 (4.16 to 4.23) | 2.44 (2.39 to 4.49) | 4.10 (4.05 to 4.16) | 3.71 (3.62 to 3.80) |
| Socio-cognitive socio-emotional gains | 4.38 (4.34 to 4.41) | 2.54 (2.48 to 2.59) | 4.18 (4.13 to 4.23) | 3.77 (3.69 to 3.86) |
| Lifestyle gains | 4.08 (4.04 to 4.13) | 2.73 (2.67 to 2.79) | 3.76 (3.70 to 3.83) | 3.20 (3.09 to 3.32) |
| AARC losses | | | | |
| Physical losses | 2.32 (2.28 to 2.36) | 2.44 (2.39 to 2.49) | 3.57 (3.51 to 3.62) | 4.20 (4.12 to 4.29) |
| Cognitive losses | 1.73 (1.69 to 1.76) | 1.87 (1.82 to 1.91) | 2.51 (2.46 to 2.56) | 3.26 (3.17 to 3.34) |
| Interpersonal losses | 1.14 (1.12 to 1.16) | 1.25 (1.22 to 1.28) | 1.79 (1.73 to 1.84) | 3.04 (2.97 to 3.11) |
| Socio-cognitive socio-emotional losses | 1.29 (1.26 to 1.32) | 1.56 (1.52 to 1.59) | 1.93 (1.88 to 1.98) | 3.25 (3.16 to 3.34) |
| Lifestyle losses | 1.53 (1.50 to 1.56) | 1.70 (1.67 to 1.74) | 2.37 (2.31 to 2.42) | 3.59 (3.51 to 3.67) |

AARC gains= Awareness of positive age-related changes. AARC losses= Awareness of negative age-related changes.
